# Swallowing-related muscle inflammation and fibrosis induced by a single dose of radiation exposure in mice

**DOI:** 10.1186/s42826-024-00199-2

**Published:** 2024-04-01

**Authors:** Shuntaro Soejima, Chia-Hsien Wu, Haruna Matsuse, Mariko Terakado, Shinji Okano, Tsuyoshi Inoue, Yoshihiko Kumai

**Affiliations:** 1 Department of Otolaryngology Head and Neck Surgery Graduate School of Biomedical Sciences, Nagasaki University, Nagasaki, Japan; 2 Department of Physiology of Visceral Function and Body Fluid, Graduate School of Biomedical Sciences, Nagasaki University, Nagasaki, Japan; 3 Department of Pathology, Nagasaki University Graduate School of Biomedical Sciences, Nagasaki University, Nagasaki, Japan

**Keywords:** Radiation-induced fibrosis, Radiation-associated dysphagia, Animal model, Mouse, Strap muscle

## Abstract

**Background:**

Although radiotherapy is commonly used to treat head and neck cancer, it may lead to radiation-associated dysphagia (RAD). There are various causes of RAD, however, the mechanism has not yet been fully identified. Currently, the only effective treatment for RAD is rehabilitation. Additionally, there are few available animal models of RAD, necessitating the development of new models to establish and evaluate RAD treatments. We hypothesize that radiation-induced neck muscle fibrosis could be one of the causes of RAD due to impairment of laryngeal elevation. Therefore, in this study, we focused on the changes in inflammation and fibrosis of the strap muscles (Sternohyoid, Sternothyroid, and Thyrohyoid muscles) after a single-dose irradiation. This research aims to provide a reference animal model for future studies on RAD.

**Results:**

Compared to control mice, those treated with 20-Gy, but not 6.7-Gy, irradiation had significantly increased tumor necrosis factor-α (TNF-α) (p < 0.01) and α-smooth muscle actin (αSMA) (*p* < 0.05) expression at 10 days and significantly increased expression levels of motif chemokine ligand-2 (CCL2), α-SMA, tumor growth factor-β1 (TGF-β1), type1 collagen, and interleukin-1β (IL-1β) (*p* < 0.05) in the muscles at 1 month by real-time PCR analysis. The results of immunohistochemistry showed that the deposition of type 1 collagen gradually increased in extracellular space after radiation exposure, and the positive area was significantly increased at 3 months compared to non-irradiated control.

**Conclusions:**

A single dose of 20-Gy irradiation induced significant inflammation and fibrosis in the strap muscles of mice at 1 month, with immunohistochemical changes becoming evident at 3 months. This cervical irradiation-induced fibrosis model holds potential for establishing an animal model for RAD in future studies.

**Level of evidence:**

N/A.

## Background

The treatment approaches for head and neck cancer (HNC) include surgery, radiotherapy (RT), and chemotherapy [1, 2]. Although RT is effective against cancers, it can damage nearby normal tissues, resulting in radiation-associated dysphagia (RAD) [3, 4]. Chemoradiotherapy can result in impaired coordination of swallowing phases due to reduced laryngeal elevation, delayed laryngeal closure, loss of tongue strength, and prolonged oral and pharyngeal time during swallowing [5, 6]. Furthermore, the peripheral and cranial nerves that innervate the swallowing musculature, including the intrinsic laryngeal musculature [7], are at risk of radiation damage, which can trigger neurogenic dysphagia due to motor and sensory deficits [8]. Laryngeal elevation, supported by the thyrohyoid and stylohyoid strap muscles in the neck, plays a crucial role in protecting the airway from aspiration of during swallowing. Cervical radiation-induced fibrosis (RIF) can lead to strap muscle fibrosis, impairing laryngeal elevation and causing RAD in HNC survivors [9]. Currently, the only effective treatment for RAD is rehabilitation [10, 11]. Moreover, no functional or histological assays are available for the evaluation of RIF of strap muscles.

In this preliminary study, we subjected mice to a single dose of irradiation to their necks, and evaluate the temporal changes in inflammation and fibrosis levels in strap muscles at three time points. By verifying the inflammation and fibrosis levels in cervical RIF model over the short term, an effective mouse model of RAD may be developed in the future.

## Methods

### Experimental animals

Thirty C57BL/6 J male mice aged 8 weeks were obtained from CLEA Japan (Tokyo, Japan) and acclimated in an animal facility for 1 week before the experiments. The mice were housed in cages under a 12-h light/dark cycle with ad libitum access to food and water. The study protocol was approved by Nagasaki University (no.: 2110131754).

### Development of the cervical RIF mouse model

**Fig. 1:**
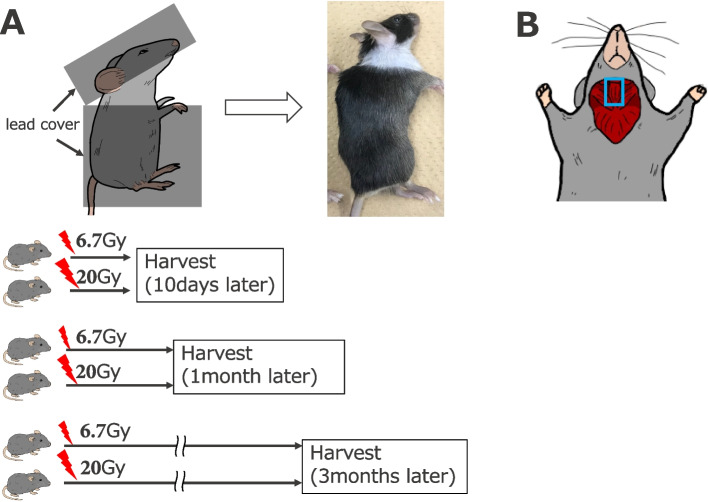
Experimental design. **A** A lead cover was used to cover the body of the mice, except for the neck. The mice received a single dose of 6.7- or 20-Gy irradiation and were sacrificed 10 days, 1 month, or 3 months thereafter. **B** Schematic showing the location of cervical neck muscles

Mice were anesthetized by intraperitoneal injection of an anesthetic reagent composed of medetomidine (0.3 mg/kg), butorphanol (5 mg/kg), and midazolam (4 mg/kg). The mice were positioned on their left side and subjected to irradiation of the anterior neck from the right side using Isovolt Titan (GE Inspection Technologies, Hürth, Germany) (upper panel in Fig. 1A). The head and trunk were covered with arched lead to prevent radiation exposure except to the neck. The mice received a single dose of 6.7- or 20-Gy irradiation (0.3937 Gy/min) and were sacrificed 10 days, 1 month, or 3 months thereafter (*n* = 6 for each condition) (lower panel in Fig. 1A).

### Gene expression analysis using quantitative real-time PCR (qPCR)

Mice were sacrificed at the indicated time point (10 days, 1 month, or 3 months) followed by isolation of the strap muscles. Total RNA was extracted from strap muscles using RNAiso (TaKaRa Bio, Inc., Tokyo, Japan) according to the manufacturer’s instructions. Then, 500 ng of total RNA was reverse-transcribed to cDNA using PrimeScript™ RT Master Mix (TaKaRa Bio, Inc.). The gene expression levels of α-smooth muscle actin (α-SMA), tumor growth factor (TGF-β), Col1, tumor necrosis factor TNF-α, C–C motif chemokine ligand 2 (CCL2), interleukin (IL)-1β, and GAPDH were measured using the CFX Connect™ Real-Time PCR Detection System (Bio-Rad Laboratories, Hercules, CA, USA) and iTaq™ Universal SYBR® Green Supermix (Bio-Rad Laboratories). Primer sequences were listed in Table 1. The expression level were normalized by internal control (GAPDH). Relative gene expression levels were represented as comparative CT (^ΔΔ^Ct)value that compare to control group.

**Table 1:** Primer sequence of the marker genes

Gene name	Forward primers	Reverse primers
Gapdh	AGGTCGGTGTGAACGGATTTG	TGTAGACCATGAGTTGAGGTCA
Tnfa	GCCTCTTCTCATTCCTGCTTG	CTGATGAGAGGGAGGCCATT
Ccl2	GACCTTAGGGCAGATGCAGT	AGCTGTAGTTTTTGTCACCAAGC
Il1b	CCTTCCAGGATGAGGACATGA	AACGTCACACACCAGCAGGTT
Acta2(aSMA)	ATTGTGCTGGACTCTGGAGATGGT	TGATGTCACGGACAATCTCACGCT
Tgfb	ACGTCACTGGAGTTGTACGG	GGGGCTGATCCCGTTGATTT
Col1a1	GAGCGGAGAGTACTGGATCG	TACTCGAACGGGAATCCATC

### Histology and immunohistochemistry

The anterior neck muscle tissues were fixed in 4% paraformaldehyde in phosphate-buffered saline (PBS; pH 7.4) immediately after sampling and embedded in paraffin for histological examination and immunohistochemistry. For morphological examination, 4 μm-thick paraffin-embedded tissues were stained with hematoxylin–eosin staining according to general protocol.

For immunohistochemistry of type 1 collagen, which was used as a fibrosis marker, the sections were deparaffinization and treated with 10 mM sodium citrate buffer pH6 (RM102-C; LSI medience) for 10 min at 120℃ for antigen retrieval. The sections were treated with 0.3% H_2_O_2_ in methanol for 15 min to inactivate endogenous peroxidase activity and then incubated with a blocking solution (5% normal goat serum) for 1 h at room temperature (RT). The sections were then incubated with the rabbit anti-type 1 collagen antibody (1:200; #72026; Cell Signaling Technology) diluted in the blocking solution for overnight at 4℃. The sections were followed by incubation with horseradish peroxidase (HRP)-conjugated goat anti-rabbit immunoglobulin antibody (P0448; Dako) diluted at 1:100 for 1 h at RT. Positive area were visualized by treating the sections with 3,3-diaminobenzidine tetrahydrochloride. Finally, after counterstaining with Mayer’s hematoxylin, the sections were dehydrated and mounted. For all specimens, negative controls were prepared following the same protocol without primary antibody.

### Histological analysis

Positive area of type 1 collagen immunostaining was evaluated by ImageJ software. Three or four different regions of each anterior neck muscle sample were randomly selected at 400X magnification with microscope (Digital Sight 10, Nikon, Tokyo, Japan). Positive area of Type1 collagen was evaluated as the number of pixels by ImageJ, and the results were expressed as a mean of each sample.

### Statistical analysis

All experiments were performed in triplicate, and the results were normalized to those of the sham control group. Data are presented as mean ± standard error of the mean (SEM). Statistical analysis was performed using GraphPad Prism 9 (GraphPad Software, Inc, San Diego, CA, USA). Normality (Kolmogorov–Smirnov test) and equality of variance (Bartlett's test) were confirmed for all the data. Those data considered to be normally distributed and with homogeneous variance were further assessed by one-way analysis of variance (ANOVA) with post-hoc Tukey for multiple comparison. For subgroup that was normally distributed but did not pass equal variance test, Welch’s ANOVA was used followed by Dunnett’s multiple comparison. For data was not normal distributed, Kruskal–Wallis test with Dunn's multiple comparison was used for non-parametric test. The differences were considered significant when *P* < 0.05.

## Results

### Experimental design of the cervical RIF mouse model

Mice exposed to a single dose of 6.7- or 20-Gy radiation were sacrificed at 10 days, 1 month, or 3 months (Fig. 1A). At 1 month, mice exposed to 20-Gy radiation showed moderate skin inflammation, ulcers, and hair loss (upper right panel in Fig. 1A); at 3 months, the skin damage had recovered. The strap muscles were isolated at the indicated time points for gene expression and histological analyses (Fig. 1B).

### Changes in inflammatory gene expression after irradiation

**Fig. 2:**
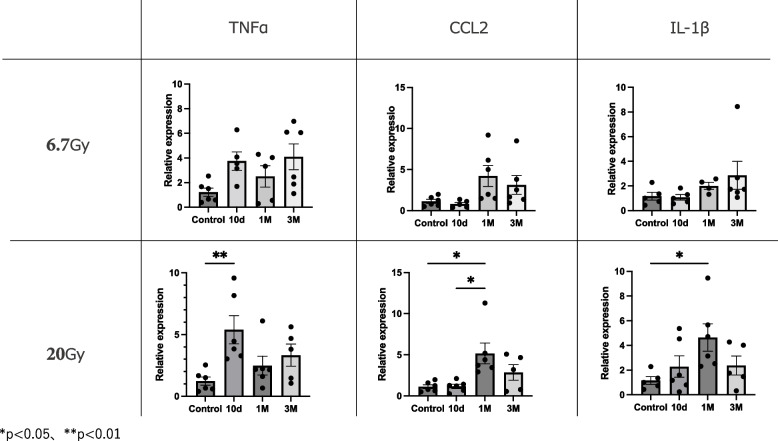
Expression levels of pro-inflammatory genes compared to controls at 10 days, 1 month, and 3 months. Relative mRNA expression levels of TNF-α, CCL2, and IL-1β in mouse strap muscles exposed to radiation. Data are presented as mean ± s.e.m. *p < 0.05, **p < 0.01

**Fig. 3:**
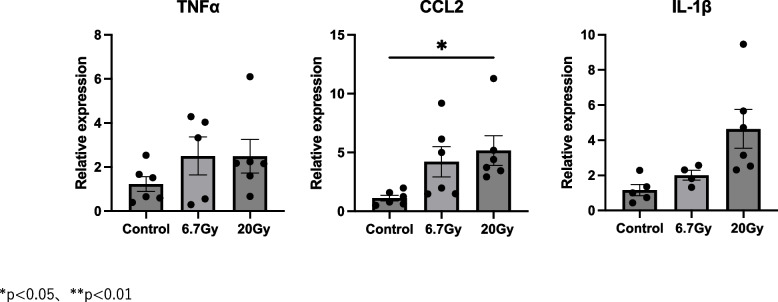
Expression levels of pro-inflammatory genes at 1 month in 6.7-Gy and 20-Gy radiation exposure groups. Relative mRNA expression levels of TNF-α, CCL2, and IL-1β in mouse strap muscles exposed to radiation. Data are presented as mean ± s.e.m. *p < 0.05, **p < 0.01

To determine the changes in expression levels of pro-inflammatory markers after 6.7- or 20-Gy radiation exposure, the mRNA levels of TNF-α, CCL2, and IL-1β were determined (Figs. 2 and 3). There were no significant differences between mice exposed to 6.7-Gy radiation and control mice (Fig. 2). The TNF-α expression level in strap muscles was significantly higher in mice exposed to 20-Gy radiation at 10 days (*p* < 0.01), but not at 1 or 3 months, compared to control mice (Fig. 2). Interestingly, the expression levels of CCL2 (p < 0.05) and IL-1β (*p* < 0.05) were significantly higher at 1 month after 20-Gy radiation exposure compared to the control group (Fig. 2), indicating time-dependent expression of inflammatory markers. At 1 month, a dose-dependent effect of irradiation on CCL2 and was observed, although the differences were only significant at 20-Gy exposure (Fig. 3).

### Changes in fibrosis-related genes after irradiation

**Fig. 4:**
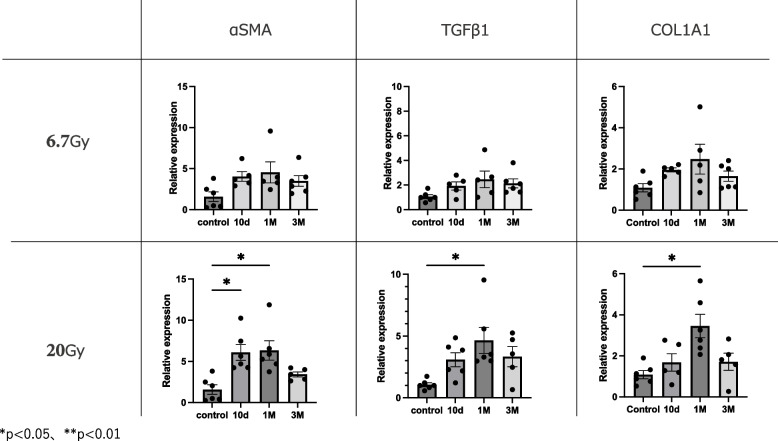
Expression levels of fibrosis-related genes compared to controls at 10 days, 1 month, and 3 months. Relative mRNA expression levels of αSMA, TGF-β1, and Col1a in mouse strap muscles exposed to radiation. Data are presented as mean ± s.e.m. *p < 0.05, **p < 0.01

**Fig. 5:**
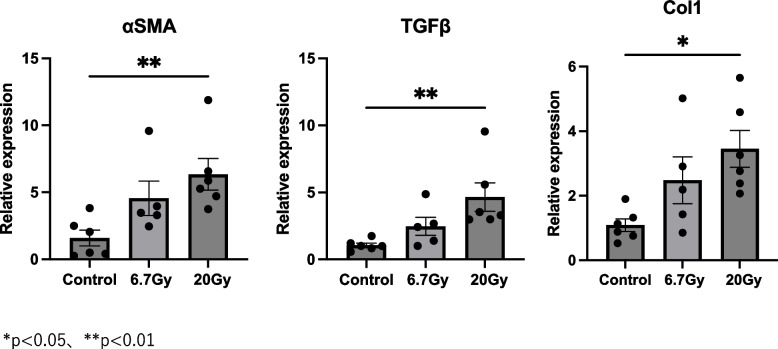
Expression levels of fibrosis-related genes at 1 month in 6.7-Gy and 20-Gy radiation exposure groups. Relative mRNA expression levels of SMA, TGF-β1, and Col1a in mouse strap muscles exposed to radiation. Data are presented as mean ± s.e.m. *p < 0.05, **p < 0.01

We analyzed the expression levels of the fibrosis-related markers α-SMA, TGF-β1, and Col1a (Figs. 4 and 5). The α-SMA mRNA expression level was significantly higher at 10 days (*p* < 0.05) and 1 month (*p* < 0.05) after 20-Gy irradiation compared to the non-irradiated strap muscles. In comparison, 20-Gy radiation significantly increased the expression levels of TGF-β1 (*p* < 0.05) and Col1a (*p* < 0.05) at 1 month. No significant differences were found in the α-SMA, TGF-β1, or Col1a mRNA levels in the 6.7-Gy-irradiated strap muscles compared to the non-irradiated muscles. In line with the changes in inflammatory markers, a dose-dependent effect of irradiation on the three fibrotic markers was observed (Fig. 5).

### Histological and immunohistochemical analysis

**Fig. 6:**
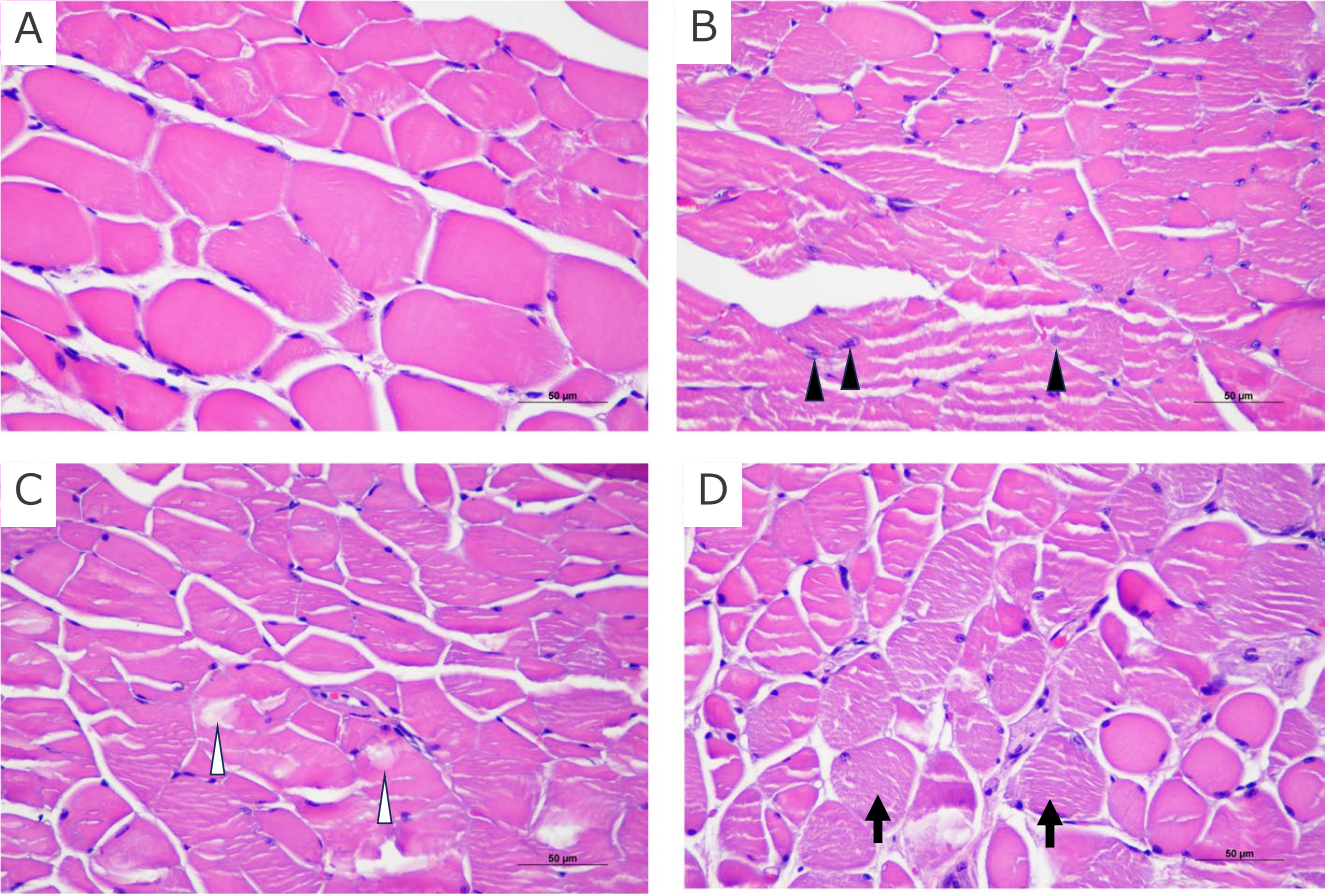
Cross section of non-irradiated and irradiated mouse anterior neck muscle stained with HE staining. **A** Control. **B** 10 days post irradiation. **C** 1 month post irradiation. **D** 3 months post irradiation

**Fig. 7:**
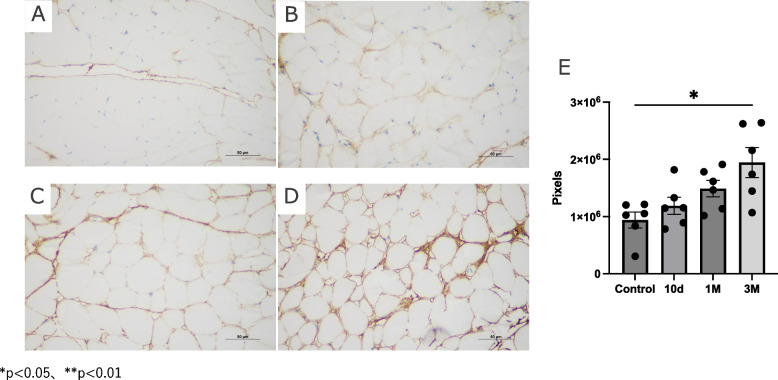
The expression of type 1 collogen in non-irradiated and irradiated mouse anterior neck muscle. Immunohistochemistry results of type 1 collogen are shown in (**A**) Control, (**B**) 10 days post irradiation, (**C**) 1 month post irradiation, and (**D**) 3 months post irradiation. **E** The bar graph shows quantitative analysis. Y axis shows the pixels of type1 collagen-positive areas. Data are presented as mean ± s.e.m

We analyzed the histological changes with HE staining and immunohistochemistry using anti-Collagen type1 antibody (Figs. 6 and 7). Non-irradiated muscle showed well-organized sarcomeres that aligned with muscle fibers (Fig. 6B). Ten days post 20-Gy radiation muscle fibers showed irregular muscle sarcomeres, which was characterized by enlarged nucleus and coarse chromatin structure compared to non-irradiated muscle (Fig. 6B, black arrowhead). One month post 20-Gy radiation muscle fibers further showed vacuolization of muscle fibers (Fig. 6C, white arrowhead), and the these irregular morphology was continuously observed in muscle 3 months post 20-Gy radiation (Fig. 6D, black arrow).

In addition, the results of immunohistochemistry showed that the deposition of type 1 collagen gradually increased in extracellular space after radiation exposure (Fig. 7A-D), and the positive area was significantly increased at 3 months compare to non-irradiated control (Fig. 7E).

## Discussion

In HNC patients, cervical RIF leads to functional problems, including dysphonia, oropharyngeal dysphagia, and chronic aspiration [12, 13]. Chronic aspiration is a life-threatening manifestation of dysphagia, affecting 30% of HNC survivors treated with irradiation [14]. RAD is not only caused by RIF of the cervical region (e.g., strap muscles, intrinsic laryngeal musculature, recurrent laryngeal nerve, and lymphatics) [7, 9, 15] but also by sensory deficits associated with cranial nerve neuropathies caused by radiation-induced hypoxia [8, 16]. Tedla et al. [7] demonstrated that swallowing dysfunction after irradiation is caused, at least partially, by a reduction in intrinsic laryngeal muscle mass and by changes in the laryngeal nerves in human models, which are involved in aspiration prevention. Starmer et al. [17] demonstrated that radiation dose to the geniohyoid rather than the constrictor muscles was more closely related to swallowing. Johns et al. [18] demonstrated that irradiated human vocal folds exhibit increased collagen transcription, with increased collagen deposition and disorganization in both the intrinsic laryngeal muscle and the superficial lamina propria. Additionally, the mouse model of irradiated vocal fold exhibits similar findings to irradiated human vocal folds and can be used to evaluate the mechanisms underlying radiation fibrosis.

RAD should be prevented to minimize the effects on the quality of life of HNC patients. Few effective treatments are available for HNC patients with RAD, including postural rehabilitation [10, 11] Krisciunas et al. [10] demonstrated that SLPs provide Manual Therapy(MT) to HNC patients during and after cancer treatment, and that reported adverse events paralleled those experienced by noncancer patients. National Cancer institute-funded prospective single-arm pilot trial called Manual Therapy for Fibrosis-Related Late Effect Dysphagia (MANTLE) is going on, which is evaluating the feasibility, safety and therapeutic potential of MT in patients with late dysphagia after radiotherapy for HNC [11]

An animal model is needed to evaluate the treatments for oropharyngeal RAD. Multiple animal models of oropharyngeal dysphagia have been developed [19], although few models of RAD are available [20–22].

Fractionated RT is typically administered for HNC to reduce side effects; this RT technique is substantially different from single-dose irradiation, and animal models should be adjusted accordingly. In previous studies, around 40–60-Gy fractionated irradiation proved sufficient for the mylohyoid muscle, which induces functional and neuronal deficits in swallowing without obvious weight loss in animals [23]. Saltman et al. [24] demonstrated that a fractionated dosing regimen was associated with lower weight loss, dehydration, and lethargy. Johns [18] demonstrated that a localized dosing protocol based on a total dose of 15-Gy administered as three 5-Gy doses at 2-week intervals prolonged the survival of animals compared to a full course of RT (15 treated animals vs. 15 controls). On the other hand, another study demonstrated the development of muscle fibrosis following administration of a single 40–90-Gy dose to hindlimbs [25]. Additionally, 75-Gy (15-Gy × 5 fractions) of radiation delivered to the mandibular area was not lethal [26]. Based on the aforementioned studies, we administered a single radiation dose of 20-Gy. The cranial motor system (i.e., tongue and swallowing-related muscles) have important differences from hindlimbs due to their different functions [27]. In the present study, we focused on the acute phase, but not the late phase, of radiation toxicity. Therefore, we used single-dose irradiation for the preliminary experiment, although multiple doses may better reflect the clinical situation. Two studies have shown that a single dose of 30- 80-Gy radiation induces skeletal muscle fibrosis in rats [28, 29]. Therefore, we used 6.7- and 20-Gy irradiation and observed the animals for 10 days and 1 month and 3 months respectively, before evaluation. Our results suggest that 20-Gy irradiation is more effective for inducing strap muscle inflammation and fibrosis compared to 6.7-Gy irradiation, as shown by the increased levels of inflammatory and fibrotic markers. King et al. [30] developed a rat model involving exposure of the submental muscle to 48-Gy fractionated radiation exposure, where this muscle is involved in the oral rather than pharyngeal phase of swallowing. The irradiated mylohyoid muscle demonstrated upregulated of TGF-β1, but not TNF-α or IL-1β, in the irradiated mylohyoid muscle compared to the non-irradiated muscle at 1 month after radiation exposure. Therefore, radiation doses < 20-Gy may not induce complete fibrosis in the strap muscles. Furthermore, the expression levels of both inflammatory and fibrotic markers in the strap muscle were significantly increased at 1 month after 20-Gy irradiation, suggesting that the strap muscles of the C57BL/6 mouse model may be sensitive to skeletal muscle and lung fibrosis, as reported previously [31].

The main mechanisms underlying radiation-induced skeletal muscles fibrosis involve DNA damage, inflammation, muscle regeneration, and fibrosis [25]. After irradiation, multiple inflammatory cytokines, including TNF-α, IL-1, and IL-6 [28, 32], are released in the early acute phase due to DNA damage, cell apoptosis, and cell necrosis. In the present study, the pro-inflammatory cytokine TNF-α exhibited increased expression, which peaked at day 10 after 20-Gy radiation. In comparison, the expression levels of inflammatory cytokines related to immune cell recruitment, i.e., CCL2 and IL-1β, were significantly increased at 1 month after 20-Gy irradiation. The time lag between changes in the expression levels of TNF-α and the remaining two cytokines suggests a phase transition from acute tissue damage to immune cell infiltration, similar to the process of muscle recovery after exercise [33]. In addition, strict regulation of the interaction between immune cells and skeletal muscles is necessary to avoid muscle fibrosis [34, 35]. Our analysis revealed significantly increased expression of fibrotic markers (α-SMA, TGF-β, and Col1a) and immunocyte recruitment markers at 1 month after 20-Gy irradiation, indicating dysregulation of the interaction between immunocytes and muscle cells. This promotes strap muscles fibrosis, as evidenced by the deposition of type1 collagen at a relatively late phase (3 months) after 20-Gy irradiation in our present study.

Finally, the expression levels of inflammatory and fibrotic markers start to decrease at 3 months compared to 1 month after irradiation, indicating the resolution of fibrosis and inflammation.

The present study evaluates pathological changes over time following radiation exposure, providing a mouse cervical RIF model to assess treatment strategies and outcomes in terms of both inflammation and fibrosis.

## Conclusions

A single dose of 20-Gy radiation can efficiently induce strap muscle inflammation and fibrosis in mice at 1 month. This cervical RIF model can be used to establish an animal model for RAD in future studies.

## Data Availability

The datasets used and/or analyzed during the current study are available from the corresponding author upon reasonable request.

## Abbreviations

α-SMA: α-Smooth muscle actin
CCL2: C-C motif chemokine ligand 2
Col1a: Type I collagen
HNC: Head and neck cancer
IL-1β: Interleukin-1β
MT: Manual therapy
RAD: Radiation-associated dysphagia
RIF: Radiation-induced fibrosis
RT: Radiotherapy
SLP: Speech language pathology
TGF-β1: Tumor growth factor-β1
TNF-α: Tumor necrosis factor
