# Correction: Swallowing-related muscle inflammation and fibrosis induced by a single dose of radiation exposure in mice

**DOI:** 10.1186/s42826-024-00227-1

**Published:** 2025-02-05

**Authors:** Shuntaro Soejima, Chia-Hsien Wu, Haruna Matsuse, Mariko Terakado, Shinji Okano, Tsuyoshi Inoue, Yoshihiko Kumai

**Affiliations:** 1 Department of Otolaryngology Head and Neck Surgery Graduate School of Biomedical Sciences, Nagasaki University, Nagasaki, Japan; 2 Department of Physiology of Visceral Function and Body Fluid, Graduate School of Biomedical Sciences, Nagasaki University, Nagasaki, Japan; 3 Department of Pathology, Nagasaki University Graduate School of Biomedical Sciences, Nagasaki University, Nagasaki, Japan

**Correction: Laboratory Animal Research (2024) 40:12** 10.1186/s42826-024-00199-2

Following publication of the original article [1], the authors reported some errors to be corrected.

**Authors’ explanation**

Since the distance from the X-ray tube to the mouse was much longer than initially expected, the actual total radiation dose turned out to be consequently smaller than we thought. With this correction, we found that irradiation dose, within the acceptable range in the actual clinical practice, could induce muscle fibrosis sufficiently in mice.

**Corrections in the article**

The corrections are reflected in the below mentioned places in the texts and figures 1, 2, 3, 4, 5.

**Table:** 

Location	Originally published text	Corrected text
Page 1, Abstract Results	72-Gy	20-Gy
Page 1, Abstract Results	24-Gy	6.7-Gy
Page 1, Abstract Conclusions	72-Gy	20-Gy
Page 2, Development of the cervical RIF mouse model	24-	6.7-
Page 2, Development of the cervical RIF mouse model	72-Gy	20-Gy
Page 2, Development of the cervical RIF mouse model	1.3948 Gy/min	0.3937 Gy/min
Page 4, Experimental design of the cervical RIF mouse model	24-	6.7-
Page 4, Experimental design of the cervical RIF mouse model	72-Gy	20-Gy
Page 4, Changes in inflammatory gene expression after irradiation	24-	6.7
Page 4, Changes in inflammatory gene expression after irradiation	72-Gy	20-Gy
Page 5, Changes in inflammatory gene expression after irradiation	72-Gy	20-Gy
Page 6, Changes in fibrosis‑related genes after irradiation	72-Gy	20-Gy
Page 6, Changes in fibrosis‑related genes after irradiation	24-Gy	6.7-Gy
Page 6, Histological and immunohistochemical analysis	72-Gy	20-Gy
Page 7, Discussion	72-Gy	20-Gy
Page 7, Discussion	24-	6.7-
Page 8, Discussion	72-Gy	20-Gy
Page 8, Conclusion	72-Gy	20-Gy

The original figure 1 was:

**Fig. 1:**
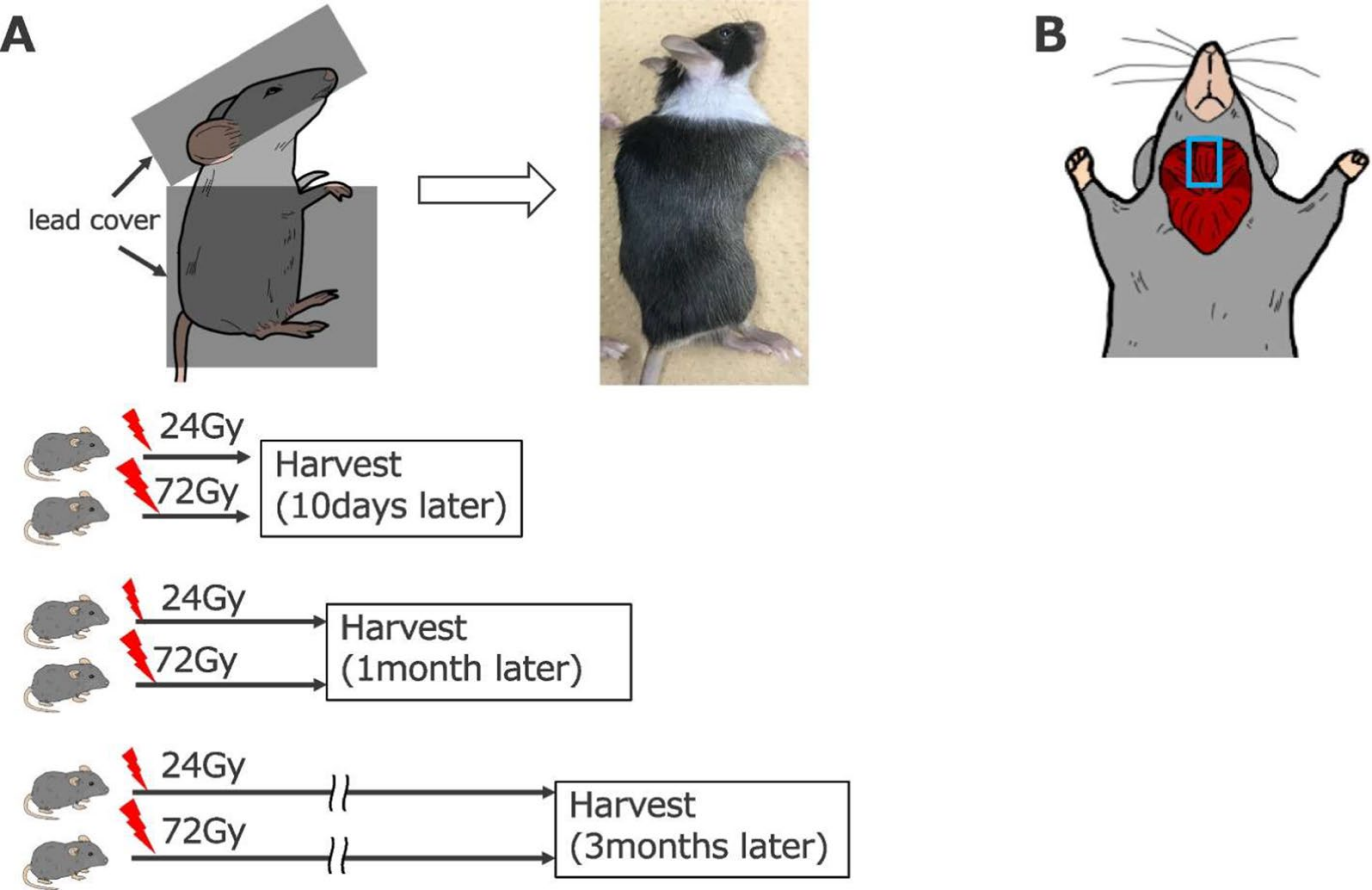
Experimental design. **A** A lead cover was used to cover the body of the mice, except for the neck. The mice received a single dose of 24- or 72-Gy irradiation and were sacrificed 10 days, 1 month, or 3 months thereafter. **B** Schematic showing the location of cervical neck muscles

The correct figure 1 should read:

**Fig. 1:**
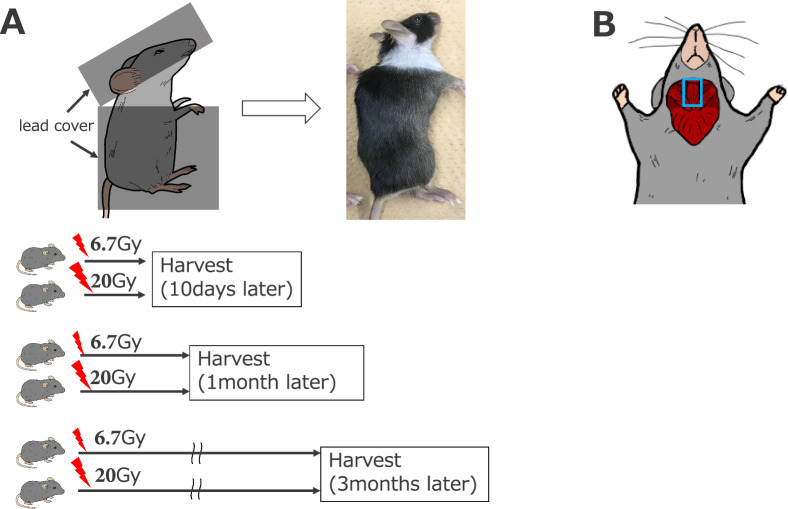
Experimental design. **A** A lead cover was used to cover the body of the mice, except for the neck. The mice received a single dose of 6.7- or 20-Gy irradiation and were sacrificed 10 days, 1 month, or 3 months thereafter. **B** Schematic showing the location of cervical neck muscles

The original figure 2 was:

**Fig. 2:**
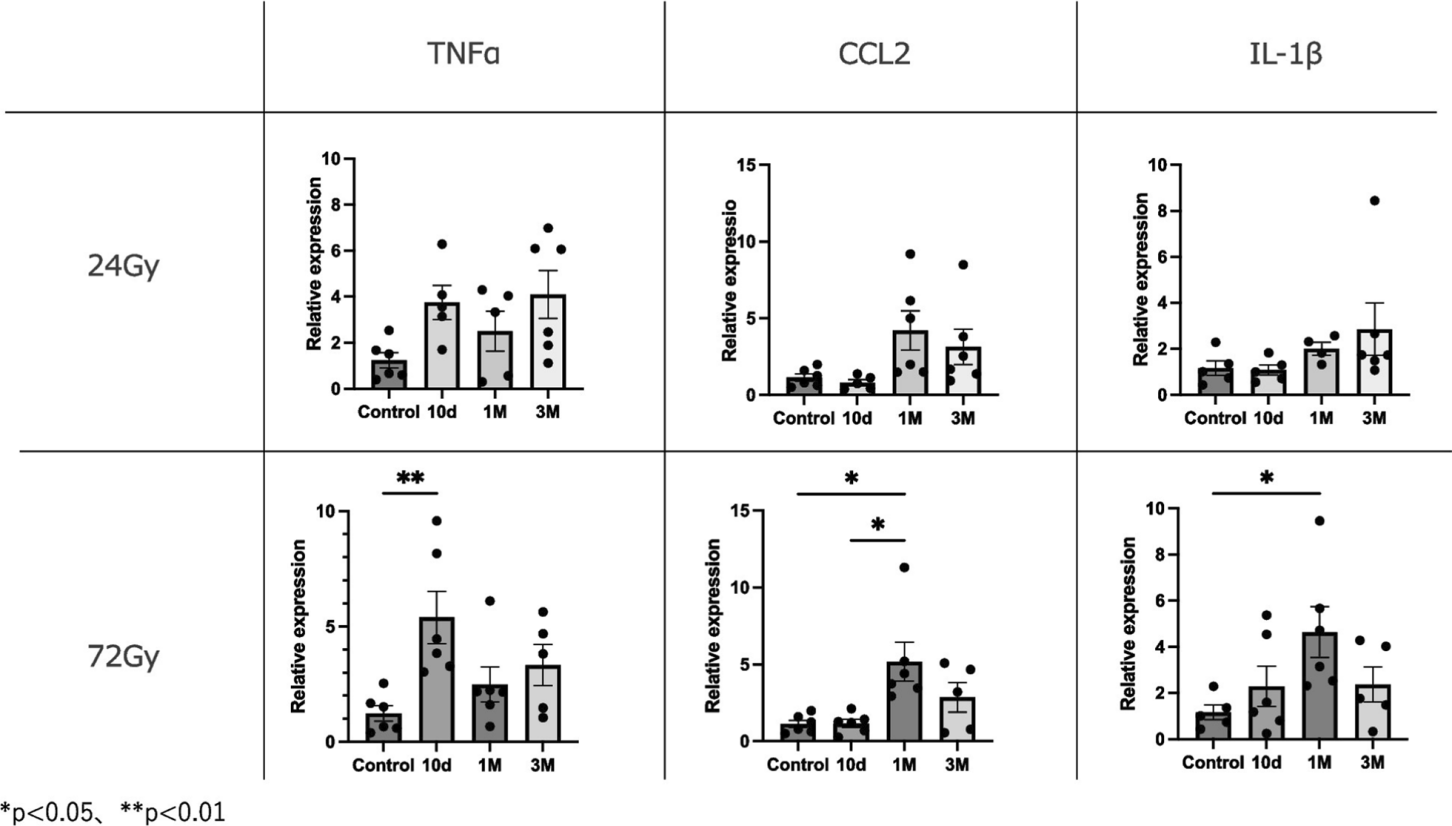
Expression levels of pro-inflammatory genes compared to controls at 10 days, 1 month, and 3 months. Relative mRNA expression levels of TNF-α, CCL2, and IL-1β in mouse strap muscles exposed to radiation. Data are presented as mean ± s.e.m. *p < 0.05, **p < 0.01

The correct figure 2 should read:

**Fig. 2:**
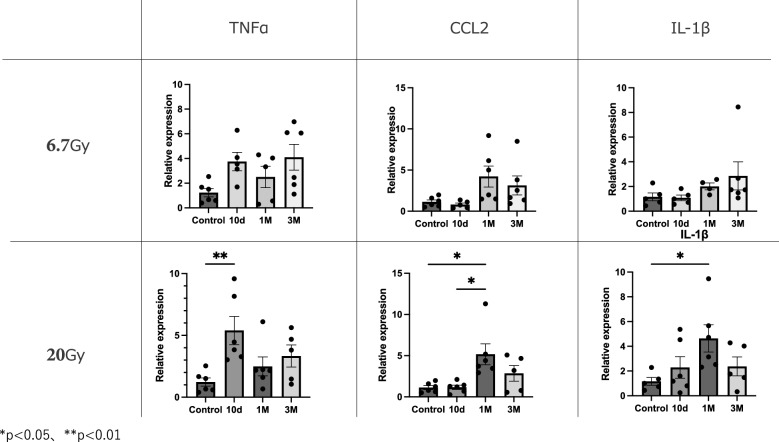
Expression levels of pro-inflammatory genes compared to controls at 10 days, 1 month, and 3 months. Relative mRNA expression levels of TNF-α, CCL2, and IL-1β in mouse strap muscles exposed to radiation. Data are presented as mean ± s.e.m. *p < 0.05, **p < 0.01

The original figure 3 was:

**Fig. 3:**
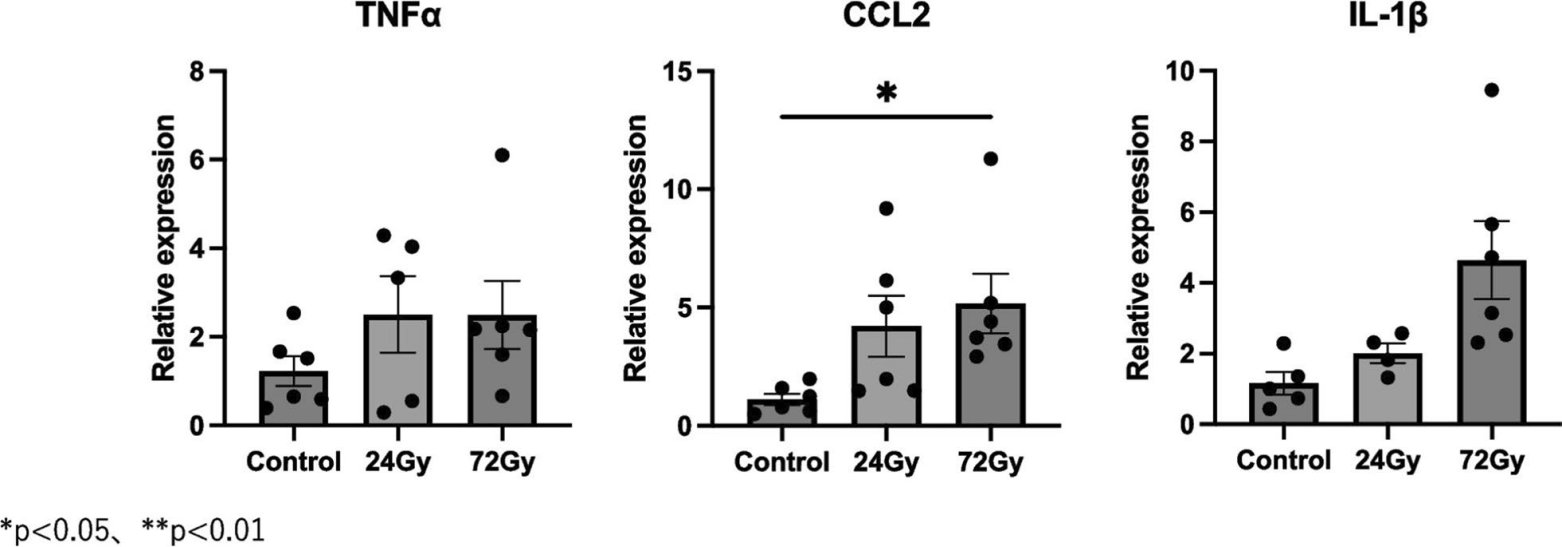
Expression levels of pro-inflammatory genes at 1 month in 24-Gy and 72-Gy radiation exposure groups. Relative mRNA expression levels of TNF-α, CCL2, and IL-1β in mouse strap muscles exposed to radiation. Data are presented as mean ± s.e.m. *p < 0.05, **p < 0.01

The correct figure 3 should read:

**Fig. 3:**
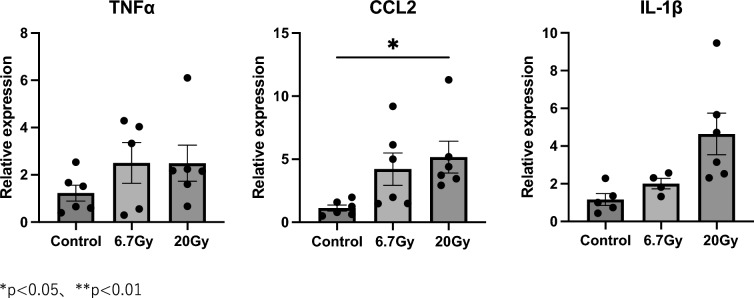
Expression levels of pro-inflammatory genes at 1 month in 6.7-Gy and 20-Gy radiation exposure groups. Relative mRNA expression levels of TNF-α, CCL2, and IL-1β in mouse strap muscles exposed to radiation. Data are presented as mean ± s.e.m. *p < 0.05, **p < 0.01

The original figure 4 was:

**Fig. 4:**
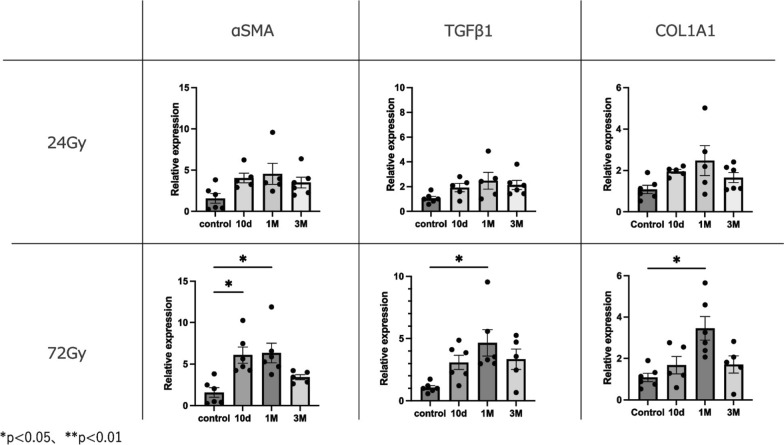
Expression levels of fibrosis-related genes compared to controls at 10 days, 1 month, and 3 months. Relative mRNA expression levels of αSMA, TGF-β1, and Col1a in mouse strap muscles exposed to radiation. Data are presented as mean ± s.e.m. *p < 0.05, **p < 0.01

The correct figure 4 should read:

**Fig. 4:**
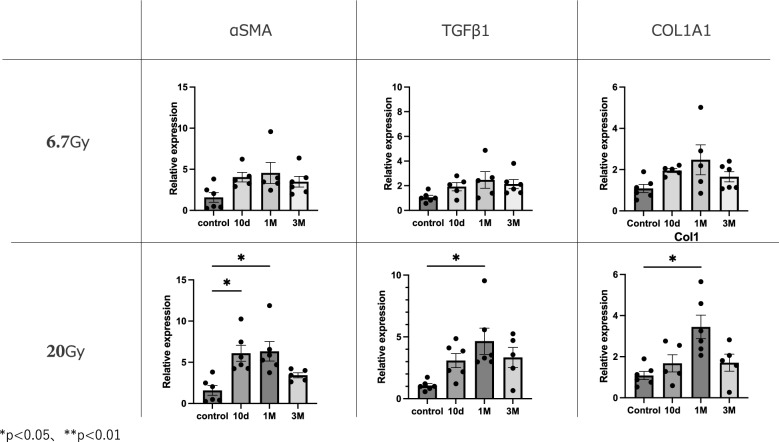
Expression levels of fibrosis-related genes compared to controls at 10 days, 1 month, and 3 months. Relative mRNA expression levels of αSMA, TGF-β1, and Col1a in mouse strap muscles exposed to radiation. Data are presented as mean ± s.e.m. *p < 0.05, **p < 0.01

The original figure 5 was:

**Fig. 5:**
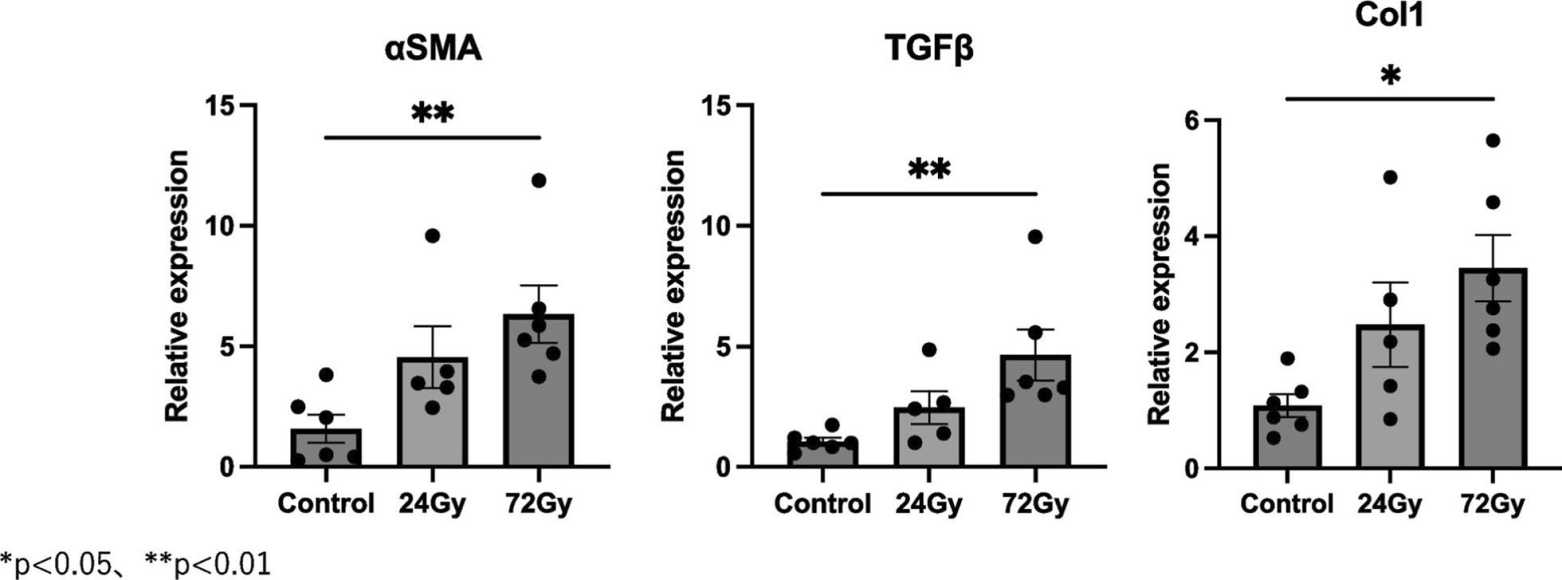
Expression levels of fibrosis-related genes at 1 month in 24-Gy and 72-Gy radiation exposure groups. Relative mRNA expression levels of SMA, TGF-β1, and Col1a in mouse strap muscles exposed to radiation. Data are presented as mean ± s.e.m. *p < 0.05, **p < 0.01

The correct figure 5 should read:

**Fig. 5:**
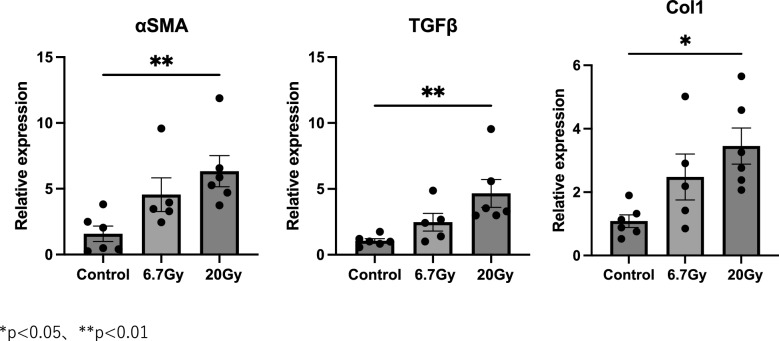
Expression levels of fibrosis-related genes at 1 month in 6.7-Gy and 20-Gy radiation exposure groups. Relative mRNA expression levels of SMA, TGF-β1, and Col1a in mouse strap muscles exposed to radiation. Data are presented as mean ± s.e.m. *p < 0.05, **p < 0.01

The original article [1] has been updated.
